# The Impact of Parental and Peer Attachment on Gaming Addiction among Out-of-School Adolescents in South Korea: The Mediating Role of Social Stigma

**DOI:** 10.3390/ijerph20010072

**Published:** 2022-12-21

**Authors:** Soyoun Kim, JongSerl Chun

**Affiliations:** Department of Social Welfare, Ewha Womans University, 52 Ewhayeodae-gil, Seodaemun-gu, Seoul 03760, Republic of Korea

**Keywords:** gaming addiction, social stigma, parenting attachment, peer attachment, out-of-school adolescents

## Abstract

Most studies on gaming addiction have targeted adolescents in schools, while studies on gaming addiction among out-of-school adolescents remain scarce. Therefore, this study investigated the influence of parental and peer attachment on gaming addiction, mediated by social stigma among Korean out-of-school adolescents. The Dropout Youth Panel Survey was used for a sample of 437 dropout adolescents. The results showed that out-of-school adolescents’ social stigma fully mediated the relationship between parental and peer attachment and gaming addiction. Parental attachment significantly predicted out-of-school adolescents’ gaming addiction by decreasing their social stigma. Peer attachment also influenced gaming addiction in out-of-school adolescents via the mediated effects of social stigma. No direct effect of parental and peer attachment on gaming addiction was found. Social stigma played an important role in decreasing levels of gaming addiction. In addition, our study revealed that the direct and total effects of parental attachment on gaming addiction were greater than the effect of peer attachment. This study empirically verified the importance of social stigma based on modified labeling theory and provides a valid mechanism to describe how Korean out-of-school adolescents develop gaming addictions. The findings suggest effective strategies for prevention and treatment for dropout adolescents in South Korea.

## 1. Introduction

Schools play an important role in adolescents’ academic and social-emotional development [1]. However, the number of dropouts has been increasing yearly in South Korea; approximately 50,000 adolescents dropped out of school in 2019 [2]. The dropout rate in 2019 was 1.0% for elementary school students, 0.8% for middle school students, and 1.7% for high school students [2]. The most frequent dropout period was in high school (60.5%), followed by middle school (26.9%) and elementary school (12.4%) [3].

In South Korea, school dropouts are seen as failures because education is highly valued, and Korean culture is defined as a collectivistic culture [4]. Korean culture promotes the idea that education for adolescents should be implemented within schools. For this reason, adolescents who drop out are at high risk of behavioral and mental health problems, such as high levels of stress [5,6], depressive symptoms [7], high levels of anxiety [8], impulsive behavior [8], and suicidal ideation [9].

Out-of-school adolescents also experience social identity crises because they tend to have few interpersonal relationships [10]. Dropouts may be excluded from the social benefits of education [11] and increased lifetime earnings [12], and are likely to experience social isolation [13] and social stigma [14,15,16]. These out-of-school adolescents often feel stigmatized and experience social discrimination [11]. Dropping out of school can also lead to neglect of parental supervision and make it challenging to spend time with friends.

Therefore, out-of-school adolescents may be drawn to cyberspace relationships and gaming addiction to avoid interpersonal problems or experience a sense of belonging [16]. As of 2016, 23.7% of out-of-school adolescents play online gambling games, as compared with only 4.4% of general students [3].

Such concerns make it essential to examine factors affecting the gaming addictions of out-of-school adolescents, to develop preventive interventions. Although the body of research on the factors influencing gaming addiction in the general student population is growing, research examining the risk factors for gaming addiction among out-of-school adolescents is very limited.

Internet gaming disorder, also called gaming addiction, is defined in the Diagnostic and Statistical Manual of Mental Disorders, 5th edition (DSM-5) as “a pattern of excessive and prolonged internet gaming that results in a cluster of cognitive and behavior symptoms, including progressive loss of control over gaming, tolerance, and withdrawal symptoms, analogous to the symptoms of substance use disorder” ([17], p. 796). The negative consequences of gaming addiction are well documented. Gaming addiction is associated with mental health issues, such as depression and anxiety [18,19], and physical ailments [18,20]. Therefore, identifying the predictive factors for gaming addiction is crucial for developing intervention strategies.

Previous studies that examined the risk factors for gaming addiction among a general student population have shown that adolescents’ psychological attributes, such as impulsivity and anxiety [21], depression [21,22], lack of parental supervision [23], interpersonal difficulties [24], and problems with peers [25] are predictors of gaming addiction. In addition, previous studies have shown that parental attachment [26], parent-child closeness [27], and peer attachment [28] are critical protective factors for gaming addiction. These risk and protective factors may also be related to gaming addiction in out-of-school adolescents [10,11,15,16].

### 1.1. Parental Attachment and Its Impact on Gaming Addiction

Out-of-school adolescents are more likely than general students to be stigmatized due to their dropout experience and to have psychosocial problems. The negative psychological emotions experienced with social stigma may be related to gaming addiction in out-of-school adolescents [15,16,29]. Therefore, in this study, we apply modified labeling theory [30,31] to structurally examine the factors influencing gaming addiction among out-of-school adolescents.

Modified labeling theory explains the stigma generated by primary deviant behavior that causes subsequent, repeated deviant behavior [30]. Adolescent delinquency can be developed by informal labeling that leads adolescents to perceive themselves as delinquent. Adolescents’ perception of self is formed by how other authorities label them and their interactions with others [30,31]. If people around them stigmatize them as troublemakers, adolescents may form negative self-perceptions, which leads to misconduct and delinquency [31]. Not only is the external stigma from authorities important; interpretation and thoughts of whether other people are stigmatizing themselves are also important [30].

Previous studies have shown that the social stigma influenced by others’ perceptions is related to gaming addiction among out-of-school adolescents [8,15,16,29]. For example, Seong [16] examined the records of 318 out-of-school youths and reported that social stigma was a predictor of gaming addiction in South Korea. Yang [8] also noted that social stigma was positively correlated with gaming addiction among Korean out-of-school adolescents, while impulsivity mediated the relationship between social stigma and gaming addiction. Furthermore, using the Youth Outside School Panel Survey from 2015 to 2017, Moon et al. [29] explored the longitudinal reciprocal relationship between gaming addiction and social stigma. They found that gaming addiction and social stigma were stable over time. They also reported that social stigma significantly predicted subsequent gaming addiction, but gaming addiction did not significantly predict subsequent social stigma.

The above review of previous studies highlights the need for further examinations of the predictors that can act as protective factors for social stigma and gaming addiction to prevent and reduce this form of addiction in out-of-school adolescents. Parental attachment [32,33,34,35] and peer attachment [35,36] are critical protective factors against social stigma. For example, Kim and Kim [34] showed that parental attachment negatively influenced social stigma, indicating that improving the quality of attachment between parents and their children effectively decreases internalized stigma among Korean out-of-school adolescents. Furthermore, Kim et al. [32] showed that informal labeling mediated the relationship between parental attachment and juvenile delinquency. Using a longitudinal survey on dropout youth, Lee et al. [35] found that parental and peer support were negatively associated with youths’ social stigma. Similarly, Yeo and Hee [36] found that parental and peer attachment was negatively correlated with social stigma among Korean dropout youths.

Previous studies on the relationship between parental attachment and gaming addiction in adolescents have revealed mixed findings. Some studies have shown that parental attachment is related to gaming addiction, indicating that higher levels of parental attachment predicted lower levels of gaming addiction [26,36,37]. Using a sample of 624 middle and high school students in South Korea, Kim and Kim [26] found a significant relationship between internet gaming addiction and parental attachment.

Furthermore, using the 2010 Korean National Data on Domestic Violence, Kim et al. [37] showed that exposure to domestic violence did not directly affect adolescents’ internet gaming addiction, but it did indirectly affect their addiction through parental attachment. Domestic violence decreased the quality of parental attachment, and low levels of parental attachment increased their gaming addiction. Using an international systematic literature review, Nielsen et al. [38] also showed that positive parenting and positive family dynamics were associated with lower rates of problematic gaming addiction among adolescents. Secure attachment with parents makes adolescents more likely to construct a positive self-concept, which helps them avoid addiction [39]. On the other hand, problematic mother–youth relationships were positively associated with youths’ later preference for online communication [40].

On the other hand, some researchers have reported that parental attachment does not directly affect gaming addiction [41,42,43,44]. For example, Throuvala et al. [42] showed that parental rejection did not directly affect internet gaming disorder and was related to internet gaming disorder only through the mediating effects of core self-evaluation. Similar results were presented by Malik et al. [41], who showed that parental attachment did not directly affect gaming addiction, but did indirectly affect it through self-control. Using a cross-lagged panel model, Teng et al. [43] examined the relationships between parent–adolescent attachment and internet gaming disorder. They reported that father and mother attachment did not predict subsequent internet gaming disorder. Yun et al. [44] also showed that parental attachment was not significantly related to internet gaming addiction in middle school students.

### 1.2. Peer Attachment and Its Impact on Gaming Addiction

During adolescence, peer relationships are very important for social-emotional development [45]. Adolescents with secure peer attachments tend to experience fewer emotional and behavioral difficulties [46]. Insecure attachment is related to emotional difficulties [45,47], delinquency [48], and conduct problems [45]. Secure attachment is positively associated with prosocial behavior [45]. Adolescents with insecure peer attachment tend to feel isolated from their peer groups and feel lonely [49]. These feelings of loneliness can lead adolescents to turn to social media [50] and online gaming as a way of escaping from real-world social relationships [16].

Previous studies have shown that peer attachment is related to gaming addiction, in showing that high levels of peer attachment decreased gaming addiction [16,43,51,52]. Yoon and Song [52] found that peer attachment was negatively related to gaming addiction using a sample of 825 middle school students in South Korea. Similar results were found in Park and Yoo’s [51] study, which examined the effect of peer attachment on gaming addiction in a sample of 318 Korean dropout youths.

Furthermore, Seong [16] examined predictors for gaming addiction in out-of-school adolescents using multiple regression analysis and reported that peer attachment and social stigma were significant predictors of gaming addiction. Similarly, Teng et al. [43] found that peer attachment had a reciprocal relationship with internet gaming disorder, indicating that peer attachment negatively predicted subsequent internet gaming disorder. Yang et al. [53] also showed that peer attachment was negatively associated with internet addiction among Chinese college students. In contrast, Soh et al. [54] reported that peer attachment did not predict internet addiction.

Adolescent gaming addiction has emerged as a serious social problem, and many studies have examined factors predicting gaming addiction. Nevertheless, most previous studies on gaming addiction have targeted adolescents attending school, while studies on gaming addiction among out-of-school adolescents remain scarce. Although out-of-school adolescents were more likely to be addicted to gaming than adolescents in school, there has been little investigation into the factors for gaming addiction among dropout adolescents. Furthermore, to our knowledge, no study has examined the mechanism connecting parental and peer attachment, social stigma, and gaming addiction. As mentioned above, social stigma may lead to gaming addiction among out-of-school adolescents [15,16,29]. Therefore, examining the predictors that might act as protective factors against social stigma among factors influencing gaming addiction and verifying their structural relationship could help to prepare prevention and treatment strategies. Parental [38,39] and peer attachment [16,43,51] are considered protective factors for gaming addiction in general youth groups. However, there is not yet consistent evidence on the relationship between parental and peer attachment and gaming addiction. Moreover, research that investigates the impact of parental and peer attachment on gaming addiction among Korean out-of-school adolescents is scarce.

**Figure 1:**
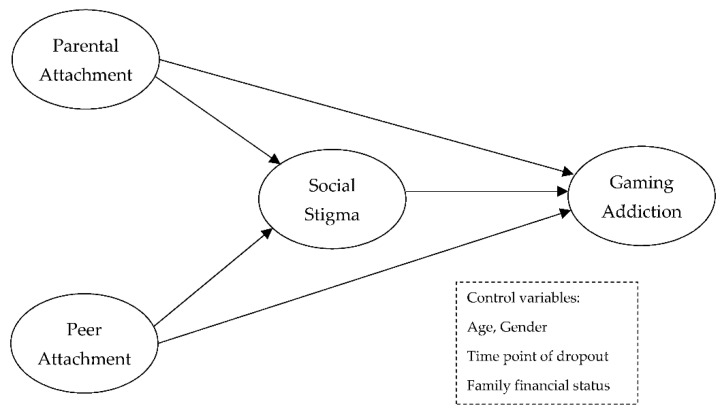
Hypothesized conceptual model.

To design effective intervention strategies for out-of-school adolescents’ gaming addiction, examining whether parental and peer attachment can function as protective factors for social stigma and gaming addiction is critical. Therefore, the present study investigated the impact of parental and peer attachment on gaming addiction among Korean out-of-school adolescents and whether this impact is mediated by social stigma. The hypothesized conceptual model is presented in Figure 1. Based on the theoretical and empirical literature, the hypotheses were as follows:

**Hypothesis** **1:** *Parental attachment has a negative effect on out-of-school adolescents’ gaming addiction.*

**Hypothesis** **2:** *Peer attachment has a negative effect on out-of-school adolescents’ gaming addiction.*

**Hypothesis** **3:** *Social stigma has a mediating effect on the relationship between parental and peer attachment and gaming addiction.*

**Hypothesis** **3a:** *Parental attachment has a negative effect on social stigma.*

**Hypothesis** **3b:** *Peer attachment has a negative effect on social stigma.*

**Hypothesis** **3c:** *Social stigma has a positive effect on out-of-school adolescents’ gaming addiction.*

## 2. Method

### 2.1. Participants

This study used data from the Dropout Youth Panel Survey (DYPS), a five-year longitudinal study that explores the life and experience of students after dropping out of school, as well as changes in values and recognition [55]. The data were collected using a prospective panel design by the National Youth Policy Institute. The DYPS established a baseline panel targeting youth who dropped out of middle and general/special-purpose high schools since July 2012. The panels were recruited through vocational institutions, alternative educational institutions, the GED institute, counseling and welfare centers, internet promotions, snowball sampling, etc. The data were collected using a 1:1 individual interview survey method. The DYPS data were collected annually from 2013 to 2017.

In the present study, we used data from wave 2 (2014), in which 442 adolescents participated. Participants with missing information were excluded, resulting in a final sample of 437 adolescents. The final sample consisted of 42.11% females and 57.89% males; the mean age was 18.31 years (SD = 0.90).

### 2.2. Measures

#### 2.2.1. Parental Attachment

Parental attachment was measured using an instrument developed by Choi et al. [56]. It comprises two subscales: emotional support and economic support. This study used the five items of the emotional support subscale. Three example items are as follows: “My parents understand and respect my feeling well,” “My parents listened to me when I was in trouble,” and “My parents always show me love and affection.” This scale was rated on a 4-point Likert scale ranging from 1 (strongly disagree) to 4 (strongly agree). Higher scores indicated higher levels of parental attachment. The Cronbach’s alpha value of the scale was 0.933.

#### 2.2.2. Peer Attachment

Peer attachment was measured with the Korean version of the Inventory of Parent and Peer Attachment [57], which was translated from and validated by the Inventory of Parent Peer Attachment [58]. This study used three items, including “My friends understand me”, “I can talk to my friends about my concerns and problems”, and “I can confide in my friends”. This scale was rated on a 4-point Likert scale ranging from 1 (strongly disagree) to 4 (strongly agree). Higher scores indicated higher levels of peer attachment. The Cronbach’s alpha value of the scale was 0.848.

#### 2.2.3. Social Stigma

Social stigma was measured using the Korean Stigmatization Scale [59], adapted from the original version by Harvey [60]. It comprises eight items scored on a 4-point Likert scale from 1 (strongly disagree) to 4 (strongly agree). The specific items presented were “I’m viewed negatively by mainstream society” and “I feel as though mainstream society views me as having a shortcoming”. Higher scores indicated a greater perception of social stigma. The Cronbach’s alpha value of the scale was 0.790.

#### 2.2.4. Gaming Addiction

Gaming addiction was measured using an instrument developed by Choi et al. [56]. It comprises eight items scored on a 4-point Likert scale from 1 (not at all) to 4 (always). Example items are as follows: “I neglect important activities to play games” and “I unsuccessfully tried to reduce my time spent on games.” Higher scores indicated a higher risk of gaming addiction. The Cronbach’s alpha value of the scale was 0.915.

### 2.3. Analysis

The analyses were conducted using SPSS 28.0 (SPSS Inc., Chicago, IL, USA) and AMOS 28.0 (SPSS Inc., Chicago, IL, USA) software. First, descriptive statistics were analyzed to provide participants’ general characteristics, parental and peer attachment, adolescents’ social stigma, and gaming addiction. Second, correlations between parental and peer attachment, adolescents’ social stigma, and gaming addiction were estimated. Third, structural equation modeling was performed to examine whether the relationship between parental and peer attachment and adolescents’ gaming addiction is mediated by social stigma and the direct relationships among parental and peer attachment, social stigma, and gaming addiction.

Bootstrapping was used to test the significance of the mediating role with 2000 random re-samples and 95% confidence intervals. Since the chi-square test is highly sensitive to sample size, we used the ratio of chi-square to the degree of freedom (X^2^/*df*), a comparative fit index (CFI), a normed fit index (NFI), and a root mean square error of approximation (RMSEA) to determine the model fit. Good fit is indicated by an X^2^/*df* value of less than 5.0 [61], CFI and NFI values of 0.9 or higher, and an RMSEA value of 0.08 or less [62].

## 3. Results

### 3.1. Descriptive Statistics

The descriptive statistics are presented in Table 1. The mean age of the participants was 18.31 years. Participants were 253 males (57.89%) and 184 females (42.11%). Most participants lived with their parents (89.02%) and perceived their family financial status as neutral (42.56%), and 36.83% of participants perceived their family financial status as insufficient. A percentage of 74.6% of participants dropped out in high school, and 25.4% of participants dropped out in middle school. Most participants (52.63%) dropped out of school because of school-related reasons, followed by alternative education reasons (26.55%), personal issues (9.15%), delinquent behaviors (9.84%), and family issues (1.83%). The mean score for parental attachment was 3.03 (SD = 0.63), and the mean score for peer attachment was 3.10 (SD = 0.58), above the middle of the scale range. Furthermore, the mean score for social stigma was 1.99 (SD = 0.43), in the middle of the scale range. Finally, the mean gaming addiction score was 1.38 (SD = 0.53), below the middle of the scale range.

### 3.2. Correlational Analysis

The bivariate correlations between study variables are presented in Table 2. The values of skewness and kurtosis of all key study variables ranged from −0.190 to 1.617 and 0.205 to 2.485, respectively. Therefore, the normal distribution of the data is acceptable, all meeting their threshold [63]. Parental and peer attachment were negatively related to adolescents’ social stigma (r = −0.353 and −0.319, respectively, *p* < 0.001), and social stigma was positively associated with gaming addiction (r = 0.143, *p* < 0.01). Moreover, parental attachment was positively associated with peer attachment (r = 0.244, *p* < 0.001). These results suggested that parental and peer attachment could be a risk factor for social stigma, and that social stigma could be a risk factor for gaming addiction among out-of-school adolescents.

### 3.3. Structural Equation Modeling

**Figure 2:**
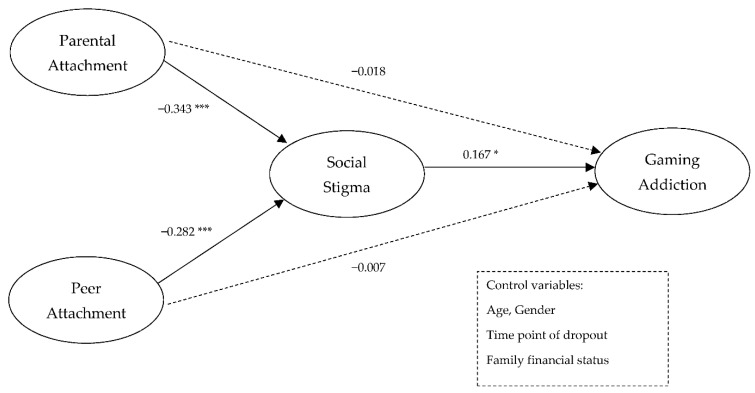
Structural equation model of parental and peer attachment, social stigma, and gaming addiction. * *p* < 0.05, *** *p* < 0.001.

To test the conceptual model, structural equation modeling analysis with latent variables was conducted by controlling age, gender, time of dropout, and family financial status. In this structural equation model, the key study variables are included as latent variables. Figure 2 shows the path analysis results of the research model investigating the influence of parental and peer attachment on adolescents’ gaming addiction mediated by social stigma. The finding of the structural equation model shows a good fit with the data [X^2^/*df* = 1.419; NFI = 0.952; TLI = 0.981; CFI = 0.9851; RMSEA = 0.031]. The paths from parental and peer attachment to gaming addiction were fully mediated by social stigma.

Table 3 shows the direct, indirect, and total effects of parental and peer attachment on adolescents’ gaming addiction via social stigma, using bootstrapping to verify the mediating effect of social stigma.

**Hypothesis** **4:** *Parental attachment was negatively associated with out-of-school adolescents’ gaming addiction. Contrary to expectations, there was a negative but non-statistically significant relation between parental attachment and gaming addiction.*

**Hypothesis** **5:** *It was hypothesized peer attachment had a negative effect on out-of-school adolescents’ gaming addiction. However, this hypothesis was rejected, by showing that there was a negative but non-statistically significant association between peer attachment and gaming addiction.*

**Hypothesis** **6:** *This hypothesis was confirmed. Social stigma mediated the relationship between parental and peer attachment and gaming addiction. Parental attachment had a negative direct effect on social stigma (β = −0.343, p < 0.01) and peer attachment had a negative effect on social stigma (β = −0.282, p < 0.01). Thus, H3a and H3b were confirmed. The results showed that social stigma had a positive effect on gaming addiction (β = 0.167, p < 0.05); thus, H3c was confirmed. These results indicate that parental and peer attachment decreased adolescents’ social stigma and that social stigma increased adolescents’ gaming addiction. Therefore, the mediating impact of social stigma was significant in the relationship between parental attachment and gaming addiction (indirect effect = −0.057, p < 0.01) and the mediating effect of social stigma was also significant in the association between peer attachment and gaming addiction (indirect effect = −0.047, p < 0.01).*

These results indicate that parental and peer attachment did not directly influence gaming addiction. Parental and peer attachment had negative direct effects on social stigma. Meanwhile, social stigma fully mediated the relationship between parental and peer attachment and gaming addiction. Parental attachment had a stronger indirect effect than peer attachment on gaming addiction through social stigma.

## 4. Discussion

In South Korea, approximately 50,000 youths dropped out of school in 2019 [2]. As of 2021, 3% of Korean adolescents were addicted to online gaming [64].

Although the number of dropouts has increased in South Korea [2], and out-of-school adolescents are more likely to be drawn to gaming addiction than general students [16], little has been known about the predictors of gaming addiction among Korean out-of-school adolescents.

The findings of the current study demonstrated that out-of-school adolescents’ social stigma fully mediated the relationship between parental and peer attachment and gaming addiction. Parental and peer attachment significantly predict out-of-school adolescents’ gaming addiction by influencing their social stigma.

The first hypothesis suggested that parental attachment would influence gaming addiction. This hypothesis was not confirmed. Parental attachment did not directly influence gaming addiction. Some studies have shown that parental attachment did not directly affect gaming addiction [41,42,43], whereas other studies indicated that parental attachment negatively affected gaming addiction [26,36,37]. The current study’s findings confirmed the assumption of modified labeling theory and the results of previous studies suggesting that parental attachment only indirectly affects gaming addiction through the mediating variable of social stigma. Out-of-school adolescents who have a secure attachment with their parents may face less social stigma from dropping out of school, which could reduce their propensity to develop problems.

The second hypothesis, which predicted a negative effect of peer attachment on gaming addiction, was rejected. Peer attachment also influenced gaming addiction in out-of-school adolescents via the mediated effects of social stigma. No direct effect of peer attachment on gaming addiction was found. This finding suggests that out-of-school adolescents who have healthy attachments with their peers are less likely to feel social stigma than those without such attachments, as indicated by previous studies [35,36]. Thus, adolescents with healthy peer relationships may be less addicted to games [8,15,16,29]. Adolescents who drop out of school tend to be disconnected from their peers and experience isolation from their peer group [13]. Previous studies have shown mixed results about the direct effect of peer attachment on gaming addiction. Some studies have indicated that peer attachment directly affected gaming addiction [16,43,51], while another study revealed an indirect effect [54]. Consistent with Soh et al.’s [54] finding, the current study revealed that peer attachment did not directly impact gaming addiction. This finding is consistent with an assumption of modified labeling theory. Adolescent delinquency can be affected by informal labeling, which is the effect of other people’s judgments on a person’s behavior [31].

Regarding the third hypothesis, the social stigma was expected to mediate the relationship between parental and peer attachment and gaming addiction of out-of-school adolescents. This hypothesis was fully confirmed. Consistent with previous research [32,33,35], parental attachment was a protective factor against social stigma. Moreover, if out-of-school adolescents have a solid attachment with their peers and are treated by their peers without prejudice, they tend to feel less social stigma and, in turn, are less addicted to games. However, out-of-school adolescents tend to have close relationships with deviant peers [65,66], who tend not to stigmatize dropping out. Such peers may influence other delinquent behaviors. Therefore, peer attachment in this population needs to be investigated. In addition, the findings regarding the effects of social stigma on gaming addiction were in line with previous studies [8,16,29], confirming that social stigma positively affects gaming addiction. Meanwhile, the finding on the relationship between parental attachment and gaming addiction informs our understanding of previous studies that presented mixed results. In the current study, social stigma played an important role in decreasing levels of gaming addiction among out-of-school adolescents. According to the Ministry of Gender Equality and Family [3], the greatest difficulty faced by out-of-school adolescents is related to social stigma, such as prejudice and disregard. Korean society promotes the negative perception that out-of-school adolescents are uniformly problematic [67]. However, adolescents may leave school for various reasons, such as to pursue specific educational interests and to save their specialties [3].

In addition, our study revealed that the direct and total effects of parental attachment on gaming addiction were greater than the effect of peer attachment. This finding suggests that parental attachment has a greater impact on reducing gaming addiction than peer attachment. In general, peer influences tend to be greater than parental influences in adolescence [68]. A study by Wu et al. [69] confirmed that peer influences were stronger than parental influences regarding gaming addiction in high school students in Taiwan. In contrast, our study showed that dropping out of school may cause more disconnection in relationships with peers than in relationships with parents; thus, the influence of parents may be greater than that of peers among out-of-school adolescents. Due to their limited interactions with peers, out-of-school adolescents’ self-concepts may be more influenced by parents than peers. Moreover, previous research has reported that parental supervision is an important protective factor against adolescent gaming addiction [70].

This study has some practical and political implications. This study emphasizes the role of parents in prevention programs. Parental education should be included in gaming addiction prevention and treatment programs, and appropriate and effective parental supervision skills should be taught in parental education programs. The findings of this study suggest that treatment programs should help adolescents build social skills to improve peer attachment and provide opportunities for them to join a new group of friends. The findings also suggest that social efforts are needed to alleviate the social stigma this population often faces. To this end, it is necessary to actively implement campaigns through the media and various social networks to inform people why out-of-school adolescents choose to develop themselves in alternative ways and how they prepare themselves to do this [67]. In addition, and more fundamentally, school education needs to consider the various needs of adolescents. Counseling support that helps out-of-school adolescents acquire skills to cope with social stigma and form positive self-perceptions should be included in gaming addiction prevention and treatment programs.

To address adolescent internet and online gaming addiction, which is a significant public health concern, the Korean government has established 18 internet-addiction prevention centers nationwide since 2002 [71]. The government also established an adolescent inpatient facility called the Korean National Center for Youth Internet Addiction Treatment in 2014 [72]. Furthermore, the City of Seoul operates six adolescent outpatient internet addiction prevention and treatment service centers [73]. However, these facilities do not currently provide tailored services for dropouts. Therefore, it is necessary to develop and provide programs designed for out-of-school adolescents to help them improve their self-esteem and deal with the social stigma of being a dropout.

Some limitations of this study (and related suggestions for future studies) should be noted. Because the current study used cross-sectional data, the causality of the assessed relationships needs to be examined using longitudinal data. Moreover, due to the lack of recent data, this study used data collected in 2014. Therefore, future studies should be conducted using more recent data. In addition, due to the limitations of secondary data analyses, only the time point of dropping out of school (middle school vs. high school) was controlled. Therefore, future studies should control the period of being out of school as a continuous variable.

## 5. Conclusions

This is the first study to investigate whether social stigma mediates parental and peer attachments’ relationships with gaming addiction among Korean out-of-school adolescents. This study empirically verified the importance of social stigma based on modified labeling theory and provides a valid mechanism to describe how Korean out-of-school adolescents develop gaming addictions. The findings suggest effective strategies for prevention and treatment of out-of-school adolescents in South Korea.

## Figures and Tables

**Table 1:** Descriptive statistics (*n* = 437).

Variables	N (%)	Mean (SD)
Age (year)		18.31 (0.90)
Sex		
Male	253 (57.89)	
Female	184 (42.11)	
Living with parents		
Yes	389 (89.02)	
No	48 (10.98)	
Self-rated family financial status		
Very insufficient	13 (2.97)	
Moderately insufficient	56 (12.81)	
Slightly insufficient	92 (21.05)	
Neutral	186 (42.56)	
Slightly sufficient	72 (16.48)	
Moderately sufficient	15 (3.43)	
Very sufficient	3 (0.69)	
Time point of school dropout		
Middle school	111 (25.40)	
High school	326 (74.60)	
Reasons for school dropout		
Individual factors	40 (9.15)	
Family factors	8 (1.83)	
School factors	230 (52.63)	
Alternative education	116 (26.55)	
Delinquent behavior	43 (9.84)	
Parental attachment		3.03 (0.63)
Peer attachment		3.10 (0.58)
Social stigma		1.99 (0.43)
Gaming addiction		1.38 (0.53)

Note: SD = standard deviation.

**Table 2:** Correlation analysis.

Variables		1	2	3	4
1.	Parental attachment	-			
2.	Peer attachment	0.244 ***	-		
3.	Social stigma	−0.353 ***	−0.319 ***	-	
4.	Gaming addiction	−0.057	−0.049	0.143 **	-
Skewness (SE)		−0.371 (0.117)	−0.647 (0.117)	−0.190 (0.117)	1.617 (0.117)
Kurtosis (SE)		0.632 (0.233)	1.893 (0.233)	0.205 (.233)	2.485 (0.233)

Note: ** *p* < 0.01, *** *p* < 0.001.

**Table 3:** The standardized direct, indirect, and total effects of parental and peer attachment on gaming addiction using 2000 bootstraps.

Variables	Direct Effect	Indirect Effect	Total Effect
Parental attachment → Social stigma	−0.343 **		−0.343 **
Parental attachment → Gaming addiction	−0.018		−0.076
Parental attachment → Social stigma → Gaming addiction		−0.057 **	
Peer attachment → Social stigma	−0.282 **		−0.282 **
Peer attachment → Gaming addiction	−0.007		−0.055
Peer attachment → Social stigma → Gaming addiction		−0.047 **	
Social stigma → Gaming addiction	0.167 *		0.167 *

Note. * *p* < 0.05, ** *p* < 0.01.

## Data Availability

The data presented in this study are available at https://www.nypi.re.kr/archive/mps/program/examinDataCode/view?menuId=MENU00226&pageNum=2&titleId=32&schType=0&schText=&firstCategory=1&secondCategory= (accessed on 11 August 2022).
